# Alcohol-Associated Liver Disease Mortality

**DOI:** 10.1001/jamanetworkopen.2025.14857

**Published:** 2025-06-11

**Authors:** Chun-Wei Pan, Yazan Abboud, Amit Chitnis, Wei Zhang, Ashwani K. Singal, Robert J. Wong

**Affiliations:** 1 Department of Internal Medicine, John H. Stroger Hospital of Cook County, Chicago, Illinois; 2 Department of Internal Medicine, Rutgers New Jersey Medical School, Newark; 3 Tuberculosis Control Section, Division of Communicable Disease Control and Prevention, Alameda County Public Health Department, San Leandro, California; 4 Gastroenterology Unit, Massachusetts General Hospital, Harvard Medical School, Boston; 5 Jewish Hospital and Trager Transplant Center, University of Louisville School of Medicine, Louisville, Kentucky; 6 Division of Gastroenterology and Hepatology, Stanford University School of Medicine, Palo Alto, California

## Abstract

**Importance:**

Alcohol-associated liver disease (ALD) is a major public health concern, accounting for one-quarter of cirrhosis-related deaths and becoming the leading indication for liver transplantation in the US, with concerning increases in mortality during and following the COVID-19 pandemic onset.

**Objective:**

To evaluate comprehensive national trends in ALD mortality in the US from 1999 to 2022, with a particular focus on disparities related to sex, race, ethnicity, and age.

**Design, Setting, and Participants:**

This retrospective cross-sectional analysis used the Centers for Disease Control and Prevention Wide Ranging Online Data for Epidemiologic Research mortality database from 1999 to 2022, examining death certificates across all 50 states and the District of Columbia. The study included individuals aged 25 years and older. Data analysis was performed from September to November 2024.

**Exposure:**

ALD mortality was identified using *International Statistical Classification of Diseases and Related Health Problems, Tenth Revision* codes (K70.xx) for both alcohol-associated hepatitis (K70.1x) and alcohol-associated cirrhosis (K70.3x).

**Main Outcomes and Measures:**

Primary outcomes included age-adjusted annual mortality rates per 100 000 population, stratified by sex, age groups (25-44, 45-64, 65-84, and ≥85 years), race and ethnicity, and geographic regions. Joinpoint regression analysis was used to calculate average annual percentage changes (AAPCs) and to identify significant trend changes.

**Results:**

In this study, a total of 436 814 ALD deaths were recorded (308 923 men [70.7%]), with ALD mortality increasing from 6.71 to 12.53 deaths per 100 000 between 1999 and 2022 and significant acceleration during 2018 to 2022 (annual percentage change [APC], 8.94%; 95% CI, 6.27% to 14.51%; *P* = .001). Women showed more rapid increases than men (AAPC, 4.29% [95% CI, 3.09% to 5.51%] vs 2.50% [95% CI, 1.51% to 3.51%]), whereas young adults (aged 25-44 years) demonstrated concerning trends (AAPC, 4.23%; 95% CI, 3.47% to 4.83%; *P* = .001). American Indian and Alaska Native populations experienced the highest mortality rates, increasing from 25.21 to 46.75 deaths per 100 000 (AAPC, 4.93%; 95% CI, 3.45% to 5.96%; *P* = .001). Alcohol-associated hepatitis mortality increased from 0.47 to 0.76 deaths per 100 000 (AAPC, 2.08%; 95% CI, 1.27% to 3.05%; *P* = .001), with women showing steeper increases than men (AAPC, 3.94% [95% CI, 2.58% to 5.46%] vs 1.56% [95% CI, 0.73% to 2.42%]). Alcohol-associated cirrhosis mortality increased from 4.09 to 9.52 deaths per 100 000 (AAPC, 4.00%; 95% CI, 3.63% to 4.40%; *P* = .001), with particularly concerning trends among women (APC from 2011 to 2022, 8.32%; 95% CI, 7.40% to 9.82%; *P* = .01) and adults aged 25 to 44 years (APC from 2018 to 2022, 19.51%; 95% CI, 15.00% to 28.53%; *P* = .001).

**Conclusions and Relevance:**

In this cross-sectional study using data from the Centers for Disease Control and Prevention Wide Ranging Online Data for Epidemiologic Research database, ALD mortality increased significantly across demographic groups, with particularly concerning trends among women, younger adults, and American Indian and Alaska Native populations. These findings highlight the urgent need for targeted public health interventions and enhanced surveillance, especially given the sustained impact of COVID-19 pandemic–related changes in alcohol consumption patterns.

## Key Points

**Question:**

What are the trends and disparities in alcohol-associated liver disease (ALD) mortality in the US from 1999 to 2022, particularly during and following the COVID-19 pandemic onset?

**Findings:**

In this cross-sectional study of 436 814 ALD-related deaths, age-adjusted mortality rates doubled from 6.71 to 12.53 deaths per 100 000 from 1999 to 2022, with significant acceleration during 2018 to 2022 (annual percentage change, 8.94%), showing disproportionate increases among women, young adults aged 25 to 44 years, and American Indian and Alaska Native populations.

**Meaning:**

The significant acceleration in ALD mortality, particularly during and following the COVID-19 pandemic onset, highlights urgent needs for targeted interventions among high-risk populations.

## Teaser

This cross-sectional study examines national trends in alcohol-associated liver disease mortality in the US from 1999 to 2022, with a particular focus on disparities by sex, race, ethnicity, and age.

## Introduction

Alcohol-associated liver disease (ALD) is a major public health concern in the US, encompassing conditions from early-stage steatosis to severe forms like alcohol-associated hepatitis (AH) and alcohol-associated cirrhosis (AC).^1,2^ ALD accounts for a major portion of liver disease morbidity and mortality, including one-quarter of cirrhosis-related deaths and more than one-third of liver disease–related hospitalizations. It has also become the leading indication for liver transplantation, imposing substantial economic burdens on health care systems.^3,4^ Recent studies^5,6,7,8,9,10,11^ have reported increasing ALD mortality rates, particularly among younger adults and women, attributed to changing alcohol consumption patterns, increasing obesity prevalence, and evolving societal norms. In addition, racial and ethnic disparities in ALD outcomes have been observed, with some minoritized groups experiencing disproportionately higher rates of severe disease and mortality.^12,13,14,15^

The COVID-19 pandemic has further exacerbated these trends. Pandemic-related stressors, such as financial insecurity and social isolation, led to increased alcohol consumption, resulting in substantial health consequences, including a 50% increase in AH admissions and substantial increases in ALD-related deaths, especially among women and younger individuals.^16,17^

Although previous studies^18,19^ have documented increasing ALD mortality rates, comprehensive analyses of COVID-19 pandemic outcomes are lacking. Better understanding these recent trends in ALD mortality will provide important information to guide public health policies and health systems in developing appropriate interventions to improve patient outcomes. In addition, elucidating disparities in ALD mortality will identify which populations need more awareness and targeted interventions. We aim to use comprehensive national mortality data to evaluate trends in ALD mortality trends in the US, with a focus on evaluating disparities in patient outcomes specifically related to sex, race, ethnicity, and age.

## Methods

This cross-sectional study used the US Centers for Disease Control and Prevention Wide Ranging Online Data for Epidemiologic Research mortality database from 1999 to 2022. We abstracted multiple cause-of-death files, which include data from death certificates across all 50 states and the District of Columbia. Each certificate provides an underlying cause of death, contributing causes, and demographic information. Our analyses focused on the underlying cause of death to identify ALD-related mortality. ALD mortality was identified using *International Statistical Classification of Diseases and Related Health Problems, Tenth Revision (ICD-10)* codes (K70.xx), with separate analyses conducted for AH (K70.1x) and AC (K70.3x).^20,21^ Details of the *ICD-10* codes used can be found in eTable in Supplement 1. This study adhered to the Strengthening the Reporting of Observational Studies in Epidemiology (STROBE) reporting guidelines to ensure the highest standard of reporting for observational studies. This study was determined to be exempt from review and informed consent by the institutional review board at Cook County Health owing to the use of deidentified, publicly available data, in accordance with 45 CFR §46.

We analyzed deaths occurring in individuals aged 25 years and older from 1999 to 2022. Age-adjusted annual mortality rates were calculated per 100 000 population and were stratified by sex (male or female), age groups (25-44, 45-64, 65-84, and ≥85 years), and race and ethnicity (American Indian or Alaska Native, Asian or Pacific Islander, Black or African American, Hispanic, and White) as classified by Centers for Disease Control and Prevention Wide Ranging Online Data for Epidemiologic Research based on death certificates using Office of Management and Budgets standards. Mortality rates were age-adjusted to the 2000 US standard population using methods suggested by the National Center for Health Statistics. This approach applies age-specific rates from our study population to the standard 2000 US population distribution across 11 age categories, producing weighted averages that allow for valid comparisons of mortality trends over time. We evaluated overall trends in age-adjusted mortality rates from 1999 to 2022 separately for ALD, AH, and AC, stratified by the aforementioned demographic characteristics.

Overall trends in annual mortality rates were presented graphically for ALD, AC, and AH, stratified by sex, race and ethnicity, age groups, and geographic region. Additional analyses were performed using Joinpoint regression methods to evaluate whether observed trends were significant during the study period. Across each time period selected by Joinpoint regression, the average annual percentage change (AAPC) and associated 95% CIs for each segment were calculated.

### Statistical Analysis

Data analysis was performed from September to November 2024. Statistical analyses were performed using R statistical software version 4.3.0 (R Project for Statistical Computing) and Joinpoint Regression Program version 5.1.0 (Surveillance Research Program, National Cancer Institute). The Joinpoint program fits data on trends into a model that detect statistically significant changes in trends. A 2-tailed *P* < .05 was used to determine statistical significance.

## Results

### ALD Mortality

**Figure 1.**
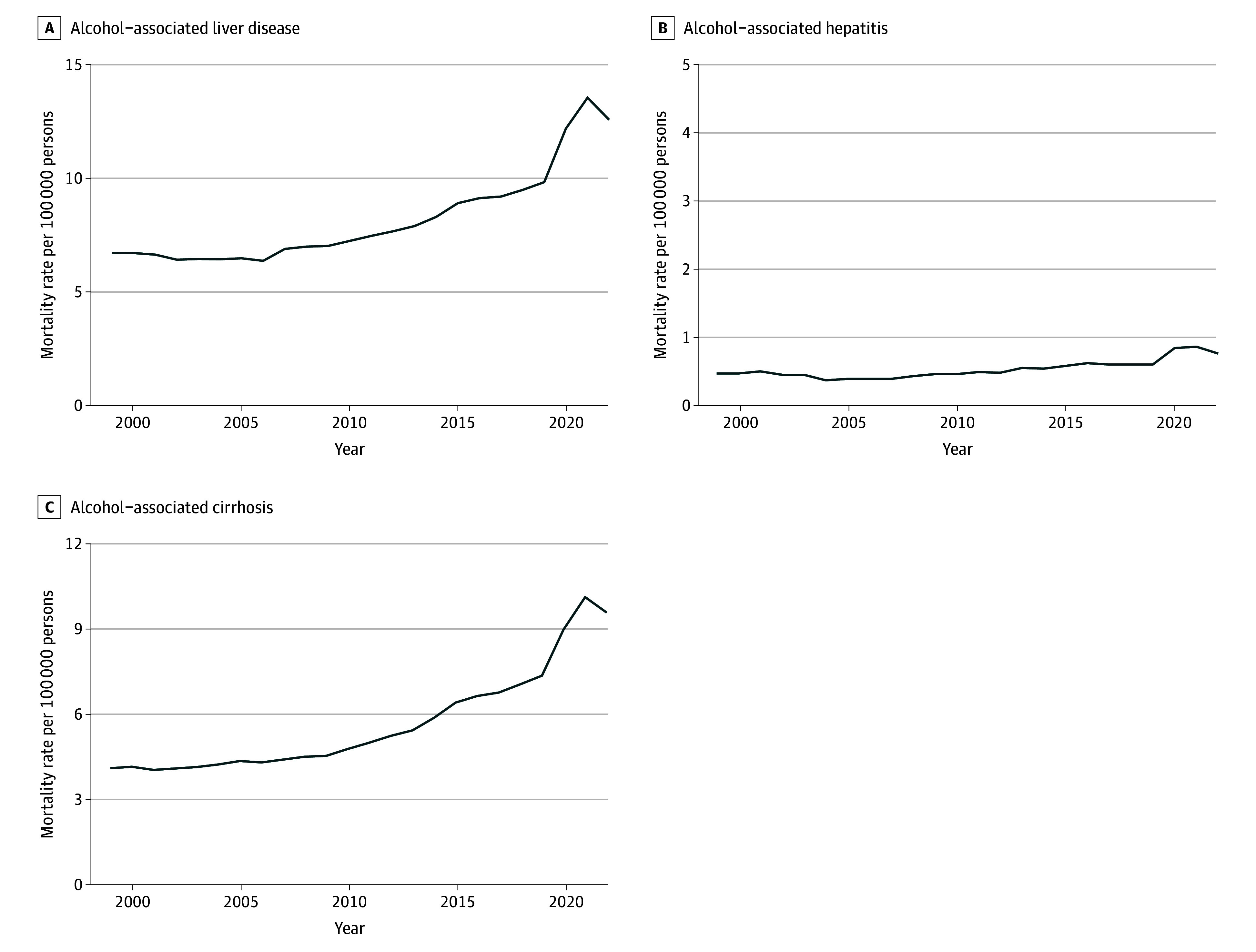
Overall Trends in Age-Adjusted Mortality Rates for Alcohol-Related Liver Disease in the US, 1999-2022 Age-adjusted mortality rates per 100 000 population are plotted against year for alcohol-associated liver disease (A), alcohol-associated hepatitis (B), and alcohol-associated cirrhosis (C).

A total of 436 814 ALD-related deaths (308 923 men [70.7%]) were recorded during the study period, with mortality rates for ALD increasing from 6.71 deaths per 100 000 (95% CI, 6.59 to 6.83 deaths per 100 000) in 1999 to 12.53 deaths per 100 000 (95% CI, 12.38 to 12.67 deaths per 100 000) in 2022 and an AAPC of 3.11% (95% CI, 2.07% to 4.16%; *P* = .001) (Figure 1). Joinpoint regression analysis revealed 3 distinct periods: 1999 to 2006, which was characterized by relative stability (annual percentage change [APC], −0.66%; 95% CI, −5.72% to 1.09%; *P* = .24); 2006 to 2018, which was marked by a significant increase (APC 3.46%, 95% CI, 2.31% to 4.59%; *P* = .003); and 2018 to 2022, which showed an even higher increase (APC, 8.94%; 95% CI, 6.27% to 14.51%; *P* = .001) (Table 1).

**Table 1.** Joinpoint Trend Segment Analyses of Age-Adjusted Mortality Rate in Alcohol-Related Liver Disease

Characteristic	Segment 1		Segment 2		Segment 3		Segment 4	
	Years	APC (95% CI)	Years	APC (95% CI)	Years	APC (95% CI)	Years	APC (95% CI)
Overall	1999-2006	−0.66 (−5.72 to 1.09)	2006-2018	3.46 (2.31 to 4.59)^a^	2018-2022	8.94 (6.27 to 14.51)^a^	NA	NA
Sex								
Male	1999-2005	−1.74 (−7.11 to 0.28)	2005-2018	2.72 (1.81 to 3.66)^a^	2018-2022	8.46 (5.73 to 14.14)^a^	NA	NA
Female	1999-2006	0.34 (−6.01 to 2.41)	2006-2018	4.75 (3.33 to 5.98)^a^	2018-2022	10.14 (7.24 to 15.84)^a^	NA	NA
Age group, y								
25-44	1999-2010	−1.32 (−5.22 to 0.14)	2010-2018	5.74 (1.61 to 9.56)^a^	2018-2022	17.69 (12.69 to 27.19)^a^	NA	NA
45-64	1999-2006	0.30 (−4.52 to 2.18)	2006-2022	4.08 (3.65 to 4.85)^a^	NA	NA	NA	NA
65-84	1999-2011	−0.13 (−1.26 to 0.72)	2011-2022	5.91 (5.22 to 6.90)^a^	NA	NA	NA	NA
≥85	1999-2010	−0.55 (−6.16 to 1.48)	2010-2022	5.11 (3.67 to 8.42)^a^	NA	NA	NA	NA
Race and ethnicity								
American Indian or Alaska Native	1999-2005	−1.66 (−8.15 to 0.81)	2005-2019	2.78 (2.20 to 3.92)^a^	2019-2022	31.70 (17.58 to 39.47)^a^	NA	NA
Asian or Pacific Islander	1999-2014	0.69 (−1.10 to 1.91)	2014-2022	7.90 (4.99 to 14.70)^a^	NA	NA	NA	NA
Black or African American	1999-2006	−6.68 (−8.83 to −4.91)^a^	2006-2012	0.59 (−8.02 to 1.85)	2012-2019	2.83 (1.62 to 4.82)^a^	2019-2022	21.45 (10.63 to 28.65)^a^
Hispanic	1999-2004	−4.38 (−11.49 to −1.17)^a^	2004-2018	1.18 (0.18 to 2.24)^a^	2018-2022	6.45 (3.66 to 11.73)^a^	NA	NA
White	1999-2005	−0.30 (−1.43 to 0.56)	2005-2019	3.57 (3.36 to 3.82)^a^	2019-2022	23.29 (19.16 to 27.43)^a^	NA	NA

Abbreviations: APC, annual percentage change; NA, not applicable.

^a^ *P* < .05.

**Figure 2.**
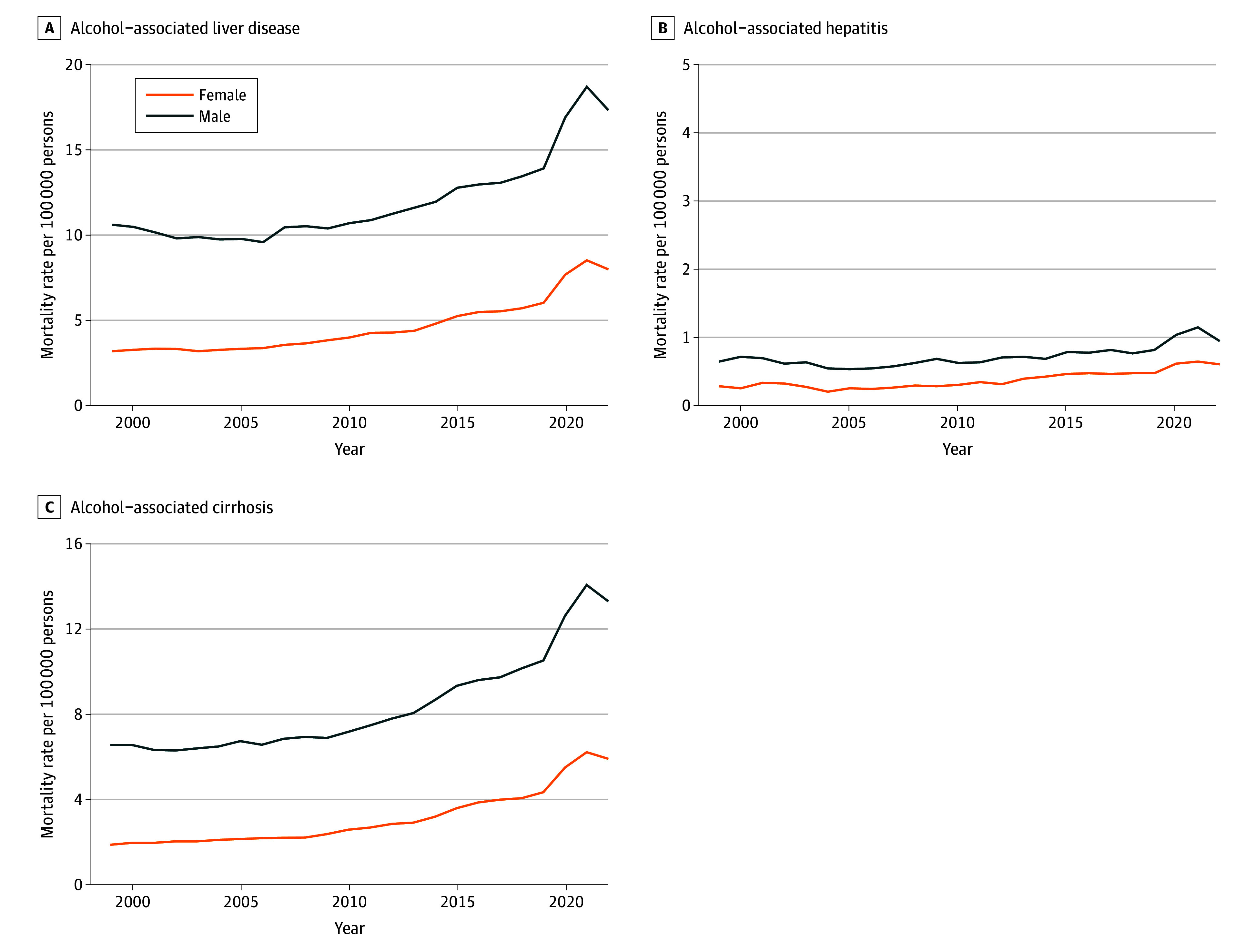
Sex-Specific Trends in Age-Adjusted Mortality Rates for Alcohol-Associated Liver Disease, 1999-2022 Age-adjusted mortality rates per 100 000 population are plotted against year, with separate lines for men and women for alcohol-associated liver disease (A), alcohol-associated hepatitis (B), and alcohol-associated cirrhosis (C).

Mortality differed according to sex throughout the study period (Figure 2). ALD-related mortality among men increased from 10.64 deaths per 100 000 (95% CI, 10.42 to 10.86 deaths per 100 000) to 17.33 deaths per 100 000 (95% CI, 17.09 to 17.57 deaths per 100 000) (AAPC, 2.50%; 95% CI, 1.51% to 3.51%; *P* = .001), whereas women experienced an even steeper increase from 3.25 deaths per 100 000 (95% CI, 3.14 to 3.37 deaths per 100 000) to 8.03 deaths per 100 000 (95% CI, 7.87 to 8.20 deaths per 100 000) (AAPC, 4.29%; 95% CI, 3.09% to 5.51%; *P* = .001).

Examination of crude mortality rate trends uncovered alarming patterns, particularly among younger adults (eFigure 1 in Supplement 1). The 25 to 44 years age group exhibited the most pronounced increase (AAPC, 4.23%; 95% CI, 3.47% to 4.83%; *P* = .001) and the most dramatic recent changes (APC from 2017 to 2022, 17.69%; 95% CI, 12.69% to 27.19%). The 45 to 64 age group maintained the highest absolute ALD mortality rates (20.66 deaths per 100 000 in 2022).

Pronounced racial and ethnic disparities in ALD mortality were observed (eFigure 2 in Supplement 1). American Indian or Alaska Native populations experienced mortality rates changing from 25.21 to 46.75 deaths per 100 000 (AAPC, 4.93%; 95% CI, 3.45% to 5.96%; *P* = .001) between 1999 and 2022, and an alarming recent acceleration from 2019 to 2022 (APC, 31.70%; 95% CI, 17.58% to 39.47%; *P* = .001). White individuals experienced a steady increase (6.63 to 13.76 deaths per 100 000; AAPC, 3.58%; 95% CI, 3.14% to 3.99%; *P* = .001), whereas Black or African American populations showed a more complex pattern, with an initial decrease followed by a sharp increase from 2019 to 2022 (APC, 21.45%; 95% CI, 10.63% to 28.35%; *P* = .03) (Table 1).

### AH Mortality

The trajectory of AH mortality from 1999 to 2022 revealed a concerning upward trend, with mortality rates increasing from 0.47 deaths per 100 000 (95% CI, 0.44 to 0.51 deaths per 100 000) to 0.76 deaths per 100 000 (95% CI, 0.72 to 0.80 deaths per 100 000), yielding an AAPC of 2.08% (95% CI, 1.27% to 3.05%; *P* = .001) (Table 2). Joinpoint regression uncovered a nuanced pattern, with an initial decline from 1999 to 2005 (APC, −5.04%; 95% CI, −16.20% to −0.61%; *P* = .03), followed by a sustained and significant increasing mortality trend from 2005 to 2022 (APC, 4.73%; 95% CI, 3.99% to 5.74%; *P* = .03).

**Table 2.** Joinpoint Trend Segment Analyses of Age-Adjusted Mortality Rate in Alcohol-Associated Hepatitis

Characteristic	Segment 1		Segment 2		Segment 3	
	Years	APC (95% CI)	Years	APC (95% CI)	Years	APC (95% CI)
Overall	1999-2005	−5.04 (−16.20 to −0.61)^a^	2005-2022	4.73 (3.99 to 5.74)^a^	NA	NA
Sex						
Female	1999-2001	13.59 (−7.95 to 34.59)	2001-2004	−13.38 (−18.18 to 15.59)	2004-2022	6.09 (1.17 to 8.73)^a^
Male	1999-2005	−5.16 (−14.99 to −1.04)^a^	2005-2022	4.04 (3.28 to 5.23)^a^	NA	NA
Age group, y^b^						
25-44	1999-2027	−4.56 (−11.54 to −0.68)^a^	2007-2022	8.42 (7.16 to 10.29)^a^	NA	NA
45-64	1999-2005	−2.41 (−12.15 to 1.73)	2005-2022	3.36 (2.62 to 5.55)^a^	NA	NA
65-84	1999-2005	−6.28 (−16.49 to −1.39)^a^	2005-2022	2.23 (1.37 to 4.21)^a^	NA	NA
Race and ethnicity^b^						
American Indian or Alaska Native	2005-2022	8.43 (6.53 to 11.23)^a^	NA	NA	NA	NA
Black or African American	1999-2005	−10.01 (−21.21 to −5.27)^a^	2005-2017	0.27 (−3.01 to 3.49)	2017-2022	12.17 (5.91 to 29.73)^a^
Hispanic	1999-2001	15.05 (−5.21 to 31.58)	2001-2005	−13.32 (−20.94 to 3.14)	2005-2022	3.39 (2.56 to 4.40)^a^
White	1999-2006	−2.50 (−11.44 to 1.23)	2006-2022	4.96 (4.17 to 6.58)^a^	NA	NA

Abbreviations: APC, annual percentage change; NA, not applicable.

^a^ *P* < .05.

^b^ The age group 85 years and older and the racial group Asian and Pacific Islander were not included due to suppression of data in accordance of Centers for Disease Control and Prevention Wide Ranging Online Data for Epidemiologic Research database reporting policy.

The sex-specific analysis demonstrated contrasting pattern, with women experiencing a more rapid increase than men. Over the study period, mortality rates for women increased from 0.28 to 0.60 deaths per 100 000 (AAPC, 3.94%; 95% CI, 2.58% to 5.46%; *P* = .001), whereas men saw an increase from 0.64 to 0.94 deaths per 100 000 (AAPC, 1.56%; 95% CI, 0.73% to 2.42%; *P* = .001) (Figure 2).

When stratified by age, adults aged 25 to 44 years recorded the highest AAPC at 3.71% (95% CI, 2.83% to 4.72%; *P* = .001), characterized by an initial decline in AH mortality from 1999 to 2007 (APC, −4.56%; 95% CI, −11.54% to −0.68%; *P* = .03), followed by a sharp reversal and increase from 2007 to 2022 (APC, 8.42%; 95% CI, 7.16% to 10.29%; *P* = .02). Other age groups also showed significant upward trends in recent years (eFigure 1 in Supplement 1).

American Indian or Alaska Native adults had the highest increase in AH mortality (AAPC, 8.43%; 95% CI, 6.53% to 11.23%; *P* = .001) from 2005 to 2022. Notably, AH mortality rates among American Indian or Alaska Native more than doubled from 1.67 deaths per 100 000 in 2010 to 3.37 deaths per 100 000 in 2022 (eFigure 2 in Supplement 1). The White population also experienced significant increases (AAPC, 2.63%; 95% CI, 1.87% to 3.51%; *P* = .001), with rates increasing from 0.45 to 0.82 deaths per 100 000 over the study period. Joinpoint analysis revealed 2 trend segments in the White population: a relatively stable period from 1999 to 2006 (APC, −2.50%; 95% CI, −11.44% to 1.23%; *P* = .68), followed by a significant increase from 2006 to 2022 (APC, 4.96%; 95% CI, 4.17% to 6.58%; *P* = .03) (Table 3).

**Table 3.** Joinpoint Trend Segment Analyses of Age-Adjusted Mortality Rate in Alcohol-Associated Cirrhosis

Characteristic	Segment 1		Segment 2		Segment 3	
	Years	APC (95% CI)	Years	APC (95% CI)	Years	APC (95% CI)
Overall	1999-2011	1.55 (0.21 to 2.53)	2011-2022	6.75 (5.98 to 7.78)^a^	NA	NA
Sex						
Female	1999-2011	2.60 (0.63 to 3.94)^a^	2011-2022	8.32 (7.40 to 9.82)^a^	NA	NA
Male	1999-2011	1.14 (−0.10 to 2.06)	2011-2022	6.02 (5.21 to 7.16)^a^	NA	NA
Age group, y						
25-44	1999-2010	−0.88 (−3.76 to 0.50)	2010-2018	7.07 (3.06 to 10.58)^a^	2018-2022	19.51 (15.00 to 28.53)^a^
45-64	1999-2009	2.32 (0.32 to 3.44)^a^	2009-2022	5.68 (5.13 to 6.56)^a^	NA	NA
65-84	1999-2010	0.05 (−0.90 to 0.87)	2010-2022	6.61 (6.11 to 7.27)^a^	NA	NA
≥85	1999-2012	1.41 (−6.18 to 3.28)	2012-2022	6.27 (4.35 to 13.77)^a^	NA	NA
Race and ethnicity						
American Indian or Alaska Native	1999-2018	3.21 (1.59 to 4.50)^a^	2018-2022	18.24 (10.72 to 34.67)^a^	NA	NA
Asian or Pacific Islander	1999-2013	1.23 (−6.50 to 3.29)	2013-2022	6.97 (4.63 to 15.72)^a^	NA	NA
Black or African American	1999-2009	−4.23 (−6.49 to −2.56)^a^	2009-2022	5.49 (4.57 to 6.75)^a^	NA	NA
Hispanic or Latino	1999-2004	−2.88 (−10.77 to 1.07)	2004-2018	2.17 (0.61 to 3.66)^a^	2018-2022	6.84 (3.76 to 12.29)^a^
White	1999-2009	1.85 (−0.59 to 2.87)	2009-2018	5.50 (2.87 to 6.64)^a^	2018-2022	9.51 (7.06 to 14.07)^a^

Abbreviations: APC, annual percentage change; NA, not applicable.

^a^ *P* < .05.

### AC Mortality

From 1999 to 2022, age-adjusted mortality rates for AC increased from 4.09 deaths per 100 000 (95% CI, 3.99 to 4.18 deaths per 100 000) to 9.52 deaths per 100 000 (95% CI, 9.40 to 9.65 deaths per 100 000), with an AAPC of 4.00% (95% CI, 3.63% to 4.40%; *P* = .001) (Figure 1C). On Joinpoint analyses, there was a small but significant increase in mortality trends from 1999 to 2011 (APC, 1.55%; 95% CI, 0.21% to 2.53%; *P* = .03), which accelerated more rapidly from 2011 to 2022 (APC, 6.75%; 95% CI, 5.98% to 7.78%; *P* = .01) (Table 3).

When stratified by sex, AC mortality from 1999 to 2022 increased from 6.59 deaths per 100 000 (95% CI, 6.41 to 6.76 deaths per 100 000) to 13.34 deaths per 100 000 (95% CI, 13.13 to 13.55 deaths per 100 000) in men and from 1.90 deaths per 100 000 (95% CI, 1.81 to 1.98 deaths per 100 000) to 5.94 deaths per 100 000 (95% CI, 5.80 to 6.08 deaths per 100 000) in women (Figure 2C). On Joinpoint regression, AC mortality among women increased by 2.60% (95% CI, 0.63% to 3.94%) per year from 1999 to 2011, and then increased more rapidly to 8.32% per year from 2011 to 2022 (APC, 8.32%; 95% CI, 7.40% to 9.82%; *P* = .01) (Table 3). For men, there was a small increase from 1999 to 2011 that was not statistically significant (APC, 1.14%; 95% CI, −0.10% to 2.06%), followed by a significant acceleration from 2011 to 2022 (APC 6.02%; 95% CI, 5.21% to 7.16%; *P* = .01).

Adults aged 45 to 64 years had the highest AC mortality rates, which increased from 6.70 deaths per 100 000 (95% CI, 6.50 to 6.91 deaths per 100 000) in 1999 to 16.09 deaths per 100 000 (95% CI, 15.82 to 16.37 deaths per 100 000) in 2022 (eFigure 1 in Supplement 1). On Joinpoint regression, among adults aged 25 to 44 years, AC mortality trends were stable from 1999 to 2010 (APC −0.88%; 95% CI, −3.76% to 0.50%; *P* = .74); however, rates started increasing from 2010 to 2018 (APC, 7.07%; 95% CI, 3.06% to 10.58%; *P* = .001) and accelerated even more rapidly from 2018 to 2022 (APC, 19.51%; 95% CI, 15.00% to 28.53%; *P* = .001) (Table 2). Other age groups also demonstrated significant increases in mortality: adults aged 45 to 64 years showed steady increases in AC from 2009 to 2022 (APC, 5.68%), whereas both older age groups (aged 65-84 and ≥85 years) experienced accelerated increases from 2010 to 2012 (Table 2).

AC mortality was highest among American Indian or Alaska Native populations, increasing from 12.11 deaths per 100 000 (95% CI, 10.21 to 14.00 deaths per 100 000) to 32.99 deaths per 100 000 (95% CI, 30.79 to 35.19 deaths per 100 000) from 1999 to 2022. As shown in Table 3, the 2018 to 2022 period revealed particularly alarming increases across multiple groups: American Indian or Alaska Native populations experienced the largest increase (APC, 18.24%; 95% CI, 10.72% to 34.67%; *P* = .03), followed by White (APC, 9.51%; 95% CI, 7.06% to 14.07%; *P* = .01) and Hispanic (APC, 6.84%; 95% CI, 3.76% to 12.29%; *P* = .02) populations. Black or African American populations showed a consistent increase from 2009 to 2022 (APC, 5.49%; 95% CI, 4.57% to 6.75%; *P* = .01). For detailed trends by race and ethnicity, see eFigure 2 in Supplement 1.

## Discussion

Our comprehensive cross-sectional analysis of ALD mortality trends in the US from 1999 to 2022 revealed several critical patterns. Overall mortality rates increased from 6.71 to 12.53 deaths per 100 000, with acceleration in recent years (2018 to 2022, AAPC, 8.94%). Although men exhibited higher mortality rates, women showed a more rapid increase (AAPC, 4.29% vs 2.50%), narrowing the sex gap. Age-stratified analysis revealed a particularly concerning trend among younger adults (aged 25 to 44 years), with the highest AAPC of 4.23%. Substantial disparities were observed across racial and ethnic groups, with American Indian or Alaska Native populations consistently showing the highest mortality rates and the most rapid increase (AAPC, 4.93%). Further detailed analysis of more severe forms of ALD showed significant increases in mortality rates for both AH (AAPC, 2.08%) and cirrhosis (AAPC, 4.00%), with particularly high increases among women and younger age groups. These findings underscore the critical nature of ALD mortality trends, highlighting disparities requiring attention.

This study extends previous observations of increasing ALD mortality in the US, with our analysis through 2022 revealing a more severe and enduring impact of the COVID-19 pandemic than previously documented.^22,23^ The accelerated increase in mortality rates from 2018 to 2022 and the persistence of elevated mortality beyond the acute phase of the pandemic suggest lasting changes in alcohol consumption patterns and health care access.^24^ This mortality trend corresponds with increasing disease incidence, as demonstrated in the Rochester Epidemiology Project, where AH incidence increased significantly, particularly among women (7-fold increase between 2000 and 2018), and increased by over 50% during the COVID-19 pandemic, while the prevalence of advanced fibrosis in patients with ALD nearly tripled from 2.2% to 6.6% between 2002 and 2016.^25,26,27^ Our analysis of both AH and AC mortality indicates concerning trends in mortality rates, likely resulting from increased alcohol consumption leading to decompensating events.^18,28^ These trends are particularly striking when compared with other liver diseases; for example, hepatitis C mortality has declined owing to effective treatments, but ALD-related deaths continue to increase,^22^ outpacing even the concerning trends seen in metabolic dysfunction-associated steatotic liver disease (AAPC, 1.98%).^29,30^ These patterns reflect a complex interplay of factors, including changing drinking behaviors, increasing obesity rates, pandemic-related health care disruptions, and broader socioeconomic stressors.^31^ The sustained increase in mortality necessitates urgent public health interventions, focusing on enhanced screening for alcohol use disorders, improved addiction treatment access, and targeted campaigns addressing high-risk drinking behaviors.

Our study provides compelling evidence of a narrowing sex gap in ALD mortality. Although men consistently exhibited higher mortality rates, women demonstrated a more rapid increase over the entire study period. Notably, this disparity was even more pronounced in the 2018 to 2022 period, with women experiencing a higher APC compared with men. These findings align with earlier studies that reported a 50% increase in alcohol-related liver injury among women from 2009 to 2015, compared with a 30% increase among men.^32,33^ Women are known to be more susceptible to ALD damage because of physiological factors such as decreased body water content and reduced gastric alcohol dehydrogenase.^33,34,35^ The accelerating female mortality rates appear driven by changing social roles, increased stress, and heightened alcohol consumption patterns.^5,36,37^ Our analysis of AH and AC mortality rates further reveals higher AAPCs among women compared with men. These patterns highlight the urgent need for sex-specific prevention and treatment strategies, including enhanced screening and alcohol cessation programs.^38,39^ The persistent narrowing of the sex gap through 2022 suggests that the pandemic’s impact on alcohol consumption and ALD progression has disproportionately affected women, necessitating targeted interventions.

ALD has been increasingly affecting younger individuals in the US. Doshi et al^36^ reported a rapid increase in AH incidence among those aged 20 to 39 years. In addition, studies have found that younger patients (aged ≤35 years) with alcohol-associated acute-on-chronic liver failure had more-severe disease, with higher rates of grades 2 to 3 acute-on-chronic liver failure compared with older patients.^18^ These concerning trends have been exacerbated during and following the COVID-19 pandemic onset. Recent data suggest a persistent elevation in alcohol consumption patterns, potentially explaining the worsening of ALD mortality trends. Our group has observed that high-risk alcohol use among US veterans has increased since the pandemic onset, with over 20% of young veterans aged 18 to 39 years reporting high-risk alcohol use in the third year following the pandemic,^40^ and these changes extend beyond veteran populations.^41^ These consumption pattern may contribute to the sustained heightened mortality rates in this age group that extend beyond year 2020 in our study. The sustained increases in high-risk drinking among young adults warrant close monitoring and targeted interventions to prevent the development and progression of ALD in this vulnerable population.

Our analysis revealed significant racial disparities in ALD mortality, with American Indian or Alaska Native populations showing the highest mortality rates and most rapid increase in recent years. This finding aligns with prepandemic data showing highest alcohol use disorder prevalence in American Indian or Alaska Native individuals^36^ and their disproportionate representation in ALD-related cirrhosis admissions (64% vs 44%-53% in other groups).^42^ The pandemic intensified these disparities, evidenced by sharp increases in AC mortality among American Indian or Alaska Native populations, while Hispanic and Black or African American individuals showed the highest increases in AH mortality. These patterns appears to align with postpandemic alcohol consumption trends showing greatest increases among minoritized racial and ethnic groups.^41,43^ Preexisting health care barriers, including higher transplant wait-list declinations and restrictive Medicaid policies,^31,44^ were combined with disproportionate COVID-19 burden.^45^ These results highlight how socioeconomic inequities, pandemic stressors, and health care disparities drive ALD mortality among minoritized racial and ethnic groups.

Our findings call for an urgent need for targeted interventions to address increasing ALD burden. Effective strategies that could be beneficial include strengthening alcohol policies through taxation and availability restriction, implementing sex-specific approaches for women,^5^ and developing culturally appropriate resources for younger adults and diverse racial and ethnic groups. Novel approaches, such as telehealth platforms and smartphone applications, may improve treatment accessibility. Research on personalized treatment, particularly sex-specific recovery pathways, remains essential.

### Limitations

This study has several limitations that should be addressed in future research. First, reliance on death certificate data and *ICD-10* codes may not accurately capture the true prevalence of ALD as a cause of death. Despite using *ICD-10* codes, the true mortality rate of AH may be underreported owing to the low positive predictive value of these codes, inaccurate coding, and misdiagnosis.^46,47^ Validation studies comparing death certificates to medical records are needed to quantify and potentially adjust for misclassification bias. Moreover, by only accounting for the underlying cause of death, we may underestimate mortality where ALD is a contributing factor. Second, the lack of individual-level data on alcohol consumption limits our ability to directly link mortality trends to changes in drinking patterns. Future studies should integrate population-level alcohol consumption data or conduct cohort studies with individual-level information to provide more insight into this relationship. It is also important to note that although we observed a steeper increase in ALD mortality as indicated by AAPC values, the 95% CIs should be considered when interpreting these trends, as some variations may be attributed to random error rather than true changes. Nevertheless, these values provide valuable understanding of the overall direction and magnitude of trends. In addition, the analysis does not account for comorbidities that may contribute to liver-related mortality, which is particularly important given the increasing prevalence of metabolic risk factors and its synergistic effect on ALD outcomes. Third, limited socioeconomic data and the need for long-term pandemic impact monitoring present additional challenges that future research must address through enhanced data collection and surveillance methods.

## Conclusions

In conclusion, mortality from ALD has increased significantly across most demographic groups in the US from 1999 to 2022, with concerning acceleration during and following the COVID-19 pandemic onset. Particularly alarming trends were observed among women, younger adults, and American Indian or Alaska Native populations. These findings demand comprehensive public health strategies focused on enhanced screening, improved addiction treatment access, and targeted interventions for high-risk groups. Long-term monitoring and research into intervention effectiveness are crucial for addressing this growing public health crisis.

## Notes

Supplement 1. **eTable.** *ICD-10* codes for alcoholic liver disease **eFigure 1.** Age-specific trends in age-adjusted mortality rates for alcohol-associated liver disease, 1999-2022 **eFigure 2.** Race/ethnicity-specific trends in age-adjusted mortality rates for alcohol-associated liver disease, 1999-2022

Supplement 2.
Data Sharing Statement
