# Supplementary material for: Alcohol-Associated Liver Disease Mortality
Source: JAMA Netw Open. 2025 Jun 11;8(6):e2514857. doi: 10.1001/jamanetworkopen.2025.14857 (PMC12159772; doi:10.1001/jamanetworkopen.2025.14857)
Supplement: Supplement 1. — eTable. ICD-10 codes for alcoholic liver disease eFigure 1. Age-specific trends in age-adjusted mortality rates for alcohol-associated liver disease, 1999-2022 eFigure 2. Race/ethnicity-specific trends in age-adjusted mortality rates for alcohol-associated liver disease, 1999-2022 [file jamanetwopen-e2514857-s001.pdf]

Supplemental Online Content

Pan CW, Abboud Y, Chitnis A, Zhang W, Singal A, Wong RJ. Alcohol-associated liver disease mortality. *JAMA Netw Open*. 2025;8(6):e2514857. doi:10.1001/jamanetworkopen.2025.14857

eTable. *ICD-10* codes for alcoholic liver disease
eFigure 1. Age-specific trends in age-adjusted mortality rates for alcohol-associated liver disease, 1999-2022
eFigure 2. Race/ethnicity-specific trends in age-adjusted mortality rates for alcohol-associated liver disease, 1999-2022

This supplemental material has been provided by the authors to give readers additional information about their work.

© 2025 Pan CW et al. *JAMA Network Open*.

eTable. *ICD-10* codes for alcoholic liver disease

Code	Description	Notes
**K70.x**	Alcoholic liver disease	Parent code encompassing all below
K70.0	Alcoholic fatty liver	
K70.1	Alcoholic hepatitis	Highlighted as specified
K70.2	Alcoholic fibrosis and sclerosis of liver	
K70.3	Alcoholic cirrhosis of liver	Highlighted as specified
K70.4	Alcoholic hepatic failure	
K70.9	Alcoholic liver disease, unspecified	

© 2025 Pan CW et al. *JAMA Network Open*.

eFigure 1. Age-specific trends in age-adjusted mortality rates for alcohol-associated liver disease, 1999-2022 a-c

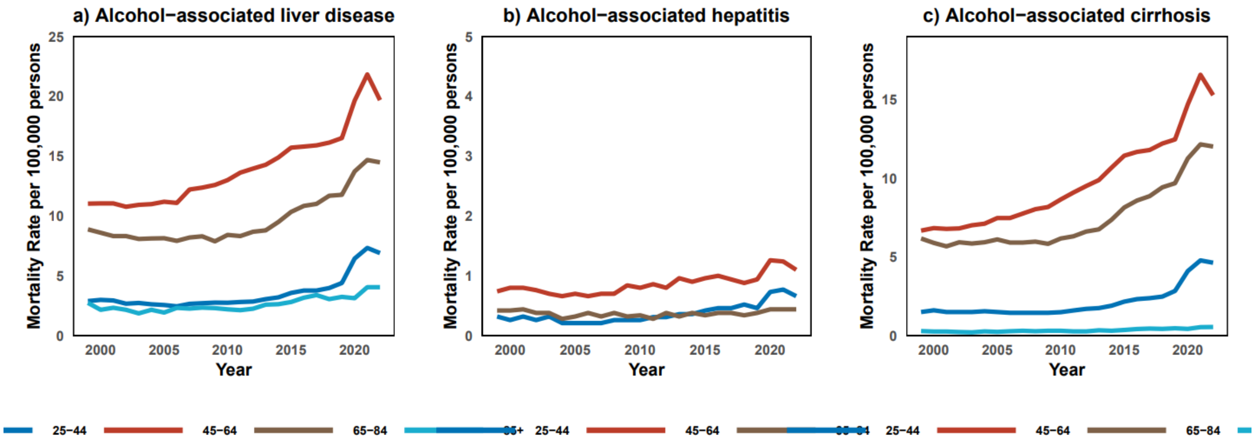

eFigure 2. Race/ethnicity-specific trends in age-adjusted mortality rates for alcohol-associated liver disease, 1999-2022, a-c

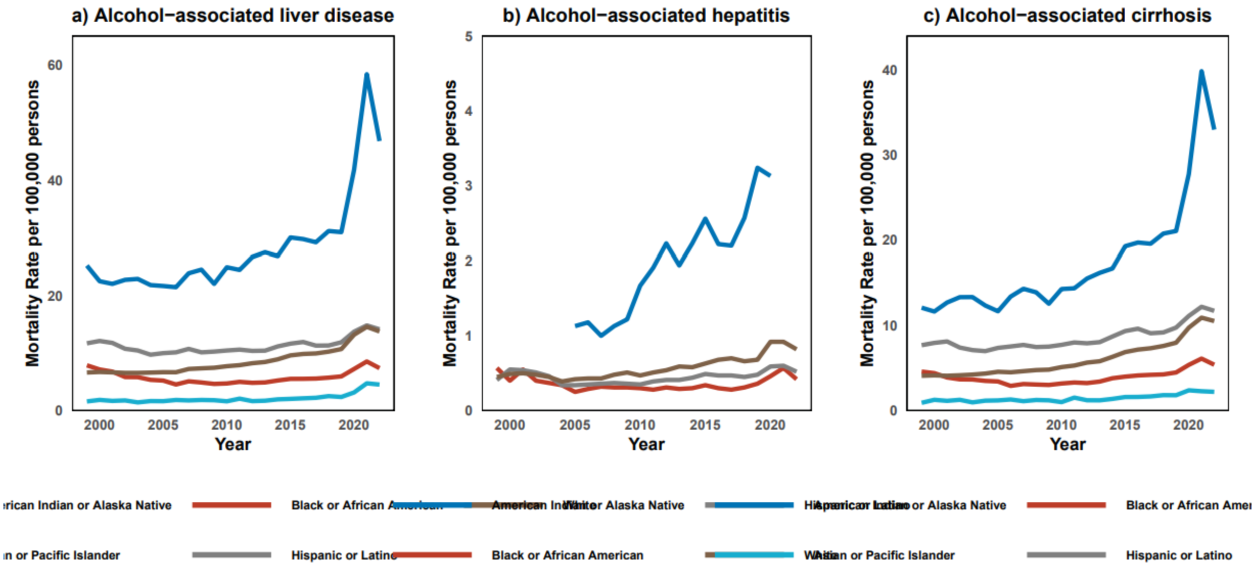

© 2025 Pan CW et al. *JAMA Network Open.*
