# Supplementary material for: Alcohol-Associated Liver Disease Mortality
Source: JAMA Netw Open. 2025 Jun 11;8(6):e2514857. doi: 10.1001/jamanetworkopen.2025.14857 (PMC12159772; doi:10.1001/jamanetworkopen.2025.14857)
Supplement: Supplement 2. — Data Sharing Statement [file jamanetwopen-e2514857-s002.pdf]

Data Sharing Statement

Pan. Alcohol-Associated Liver Disease Mortality. *JAMA Netw Open*. Published June 11, 2025.
doi:10.1001/jamanetworkopen.2025.14857

Data
Data available: Yes
Data types: Data (not involving human participants)
How to access data: https://wonder.cdc.gov/mcd.html
When available: With publication

Supporting Documents
Document types: None

Additional Information
Who can access the data: anyone requesting the data
Types of analyses: for repurpose of analyses
Mechanisms of data availability: with a signed data access agreement
